# Correction: Hybrid Dysgenesis in *Drosophila simulans* Associated with a Rapid Invasion of the *P*-Element

**DOI:** 10.1371/journal.pgen.1006058

**Published:** 2016-05-11

**Authors:** Tom Hill, Christian Schlötterer, Andrea J. Betancourt

The following information is missing from the Funding section: CS is funded through the ERC grant ArchAdapt.
