# Supplementary figures and images for: Hybrid Dysgenesis in Drosophila simulans Associated with a Rapid Invasion of the P-Element
Source: PLoS Genet. 2016 Mar 16;12(3):e1005920. doi: 10.1371/journal.pgen.1005920 (PMC4794157; doi:10.1371/journal.pgen.1005920)

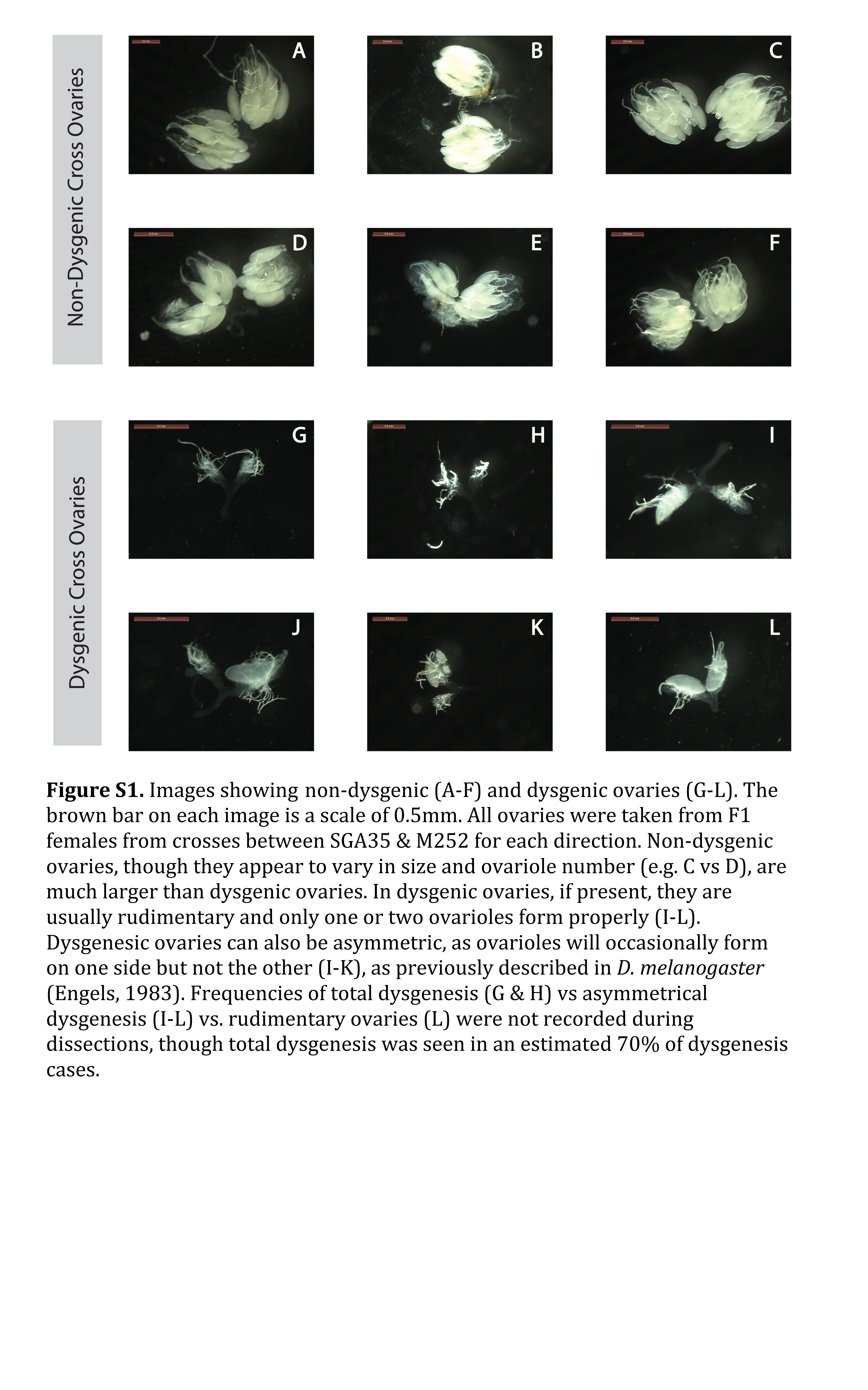

Supplement: S1 Fig — Images showing non-dysgenic (A-F) and dysgenic ovaries (G-L). The brown bar on each image is a scale of 0.5mm. All ovaries were taken from F1 females from crosses between SGA35 & M252 for each direction. Non-dysgenic ovaries, though they appear to vary in size and ovariole number (e.g. C vs. D), are much larger than dysgenic ovaries. In dysgenic ovaries, if present, they are usually rudimentary and only one or two ovarioles form properly (I-L). Dysgenic ovaries can also be asymmetric, as ovarioles will occasionally form on one side but not the other (I-K), as previously described in D. melanogaster [25]. Frequencies of total dysgenesis (G & H) vs. asymmetrical dysgenesis (I-L) vs. rudimentary ovaries (L) were not recorded during dissections, though total dysgenesis was seen in an estimated 70% of dysgenesis cases. (TIF) [file pgen.1005920.s001.tif]
