# Supplementary material for: Hybrid Dysgenesis in Drosophila simulans Associated with a Rapid Invasion of the P-Element
Source: PLoS Genet. 2016 Mar 16;12(3):e1005920. doi: 10.1371/journal.pgen.1005920 (PMC4794157; doi:10.1371/journal.pgen.1005920)
Supplement: S2 Fig — Bar plots show the fraction of dysgenic offspring for both directions of each set of reciprocal crosses. Bars are coloured red when a significant difference between each reciprocal cross is found (Fisher’s Exact Test, p < 0.05). A) Initial crosses between Georgia (2009) and Madagascar (2004) flies at 29° C, with the fraction of dysgenic F1 females shown when the Georgia strain is paternal (positive direction) or maternal (negative direction). B) A subset of the crosses in panel A were repeated at 25°C, a non-dysgenic temperature in D. melanogaster; repeated crosses are shown in the same position as in panel A. C) Fraction of dysgenic offspring from crosses within and between Georgia and Florida lines at 29°C. The first strain named in the cross is the paternal strain in the positive direction and the maternal strain in the negative direction. D) Fraction of dysgenic offspring from crosses within and between Madagascar lines at 29°C. The first strain named in the cross is the paternal strain in the positive direction and the maternal strain in the negative direction. E) Fraction of dysgenic offspring from crosses between Georgia and Madagascar lines at 29C after curing of Wolbachia with tetracycline hydrachloride for 2 generations. F) Fraction of dysgenic offspring from crosses between Georgia and Madagascar lines at 29C before curing. (PDF) [file pgen.1005920.s002.pdf]

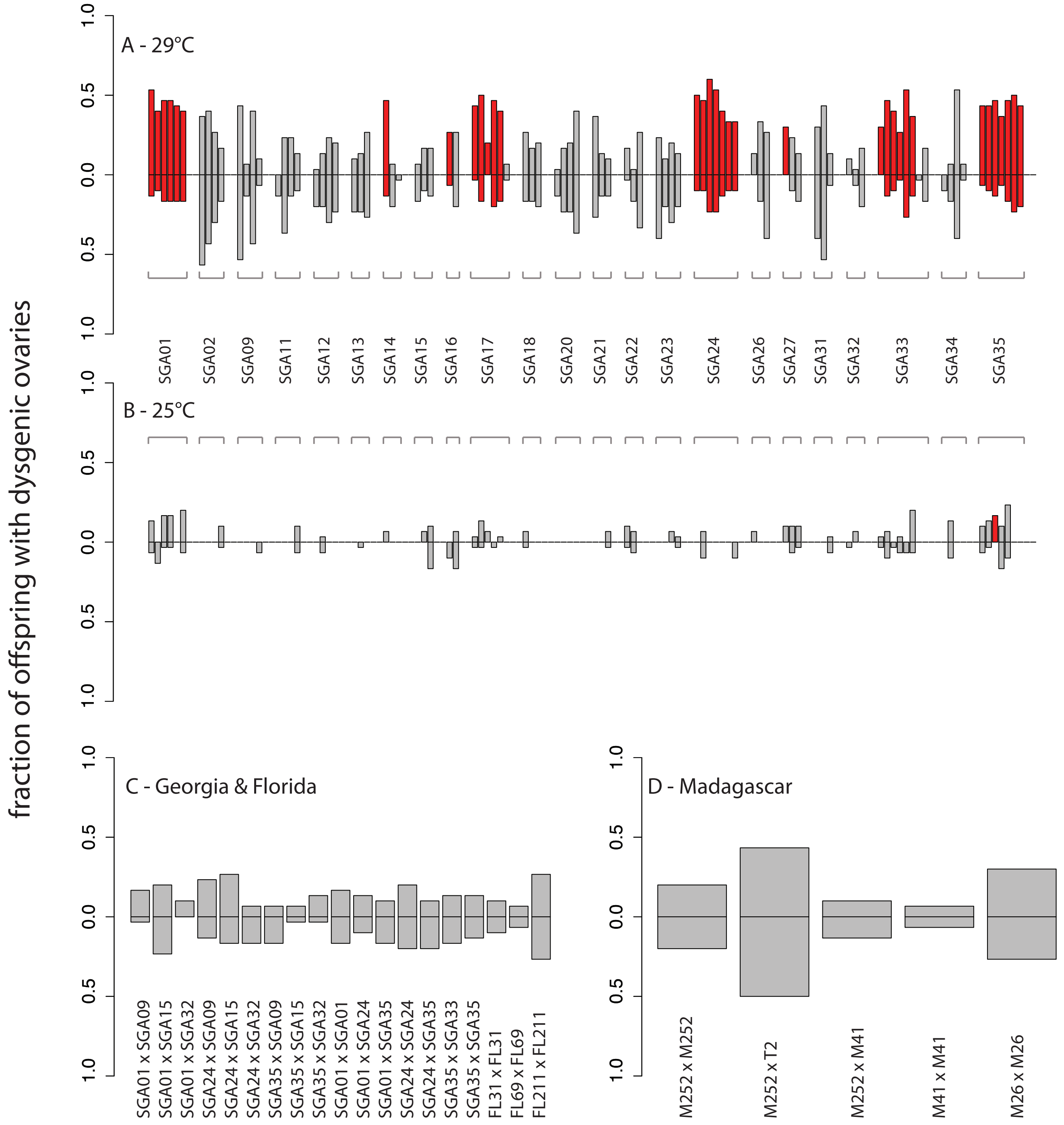
fraction of offspring with dysgenic ovaries
A - 29°C
B - 25°C
C - Georgia & Florida
D - Madagascar
1.0
0.5
0.0
0.5
1.0
SGA01
SGA02
SGA09
SGA11
SGA12
SGA13
SGA14
SGA15
SGA16
SGA17
SGA18
SGA20
SGA21
SGA22
SGA23
SGA24
SGA26
SGA27
SGA31
SGA32
SGA33
SGA34
SGA35
SGA01 x SGA09
SGA01 x SGA15
SGA01 x SGA32
SGA24 x SGA09
SGA24 x SGA15
SGA24 x SGA32
SGA35 x SGA09
SGA35 x SGA15
SGA35 x SGA32
SGA01 x SGA01
SGA01 x SGA24
SGA01 x SGA35
SGA24 x SGA24
SGA24 x SGA35
SGA35 x SGA33
SGA35 x SGA35
FL31 x FL31
FL69 x FL69
FL211 x FL211
M252 x M252
M252 x T2
M252 x M41
M41 x M41
M26 x M26

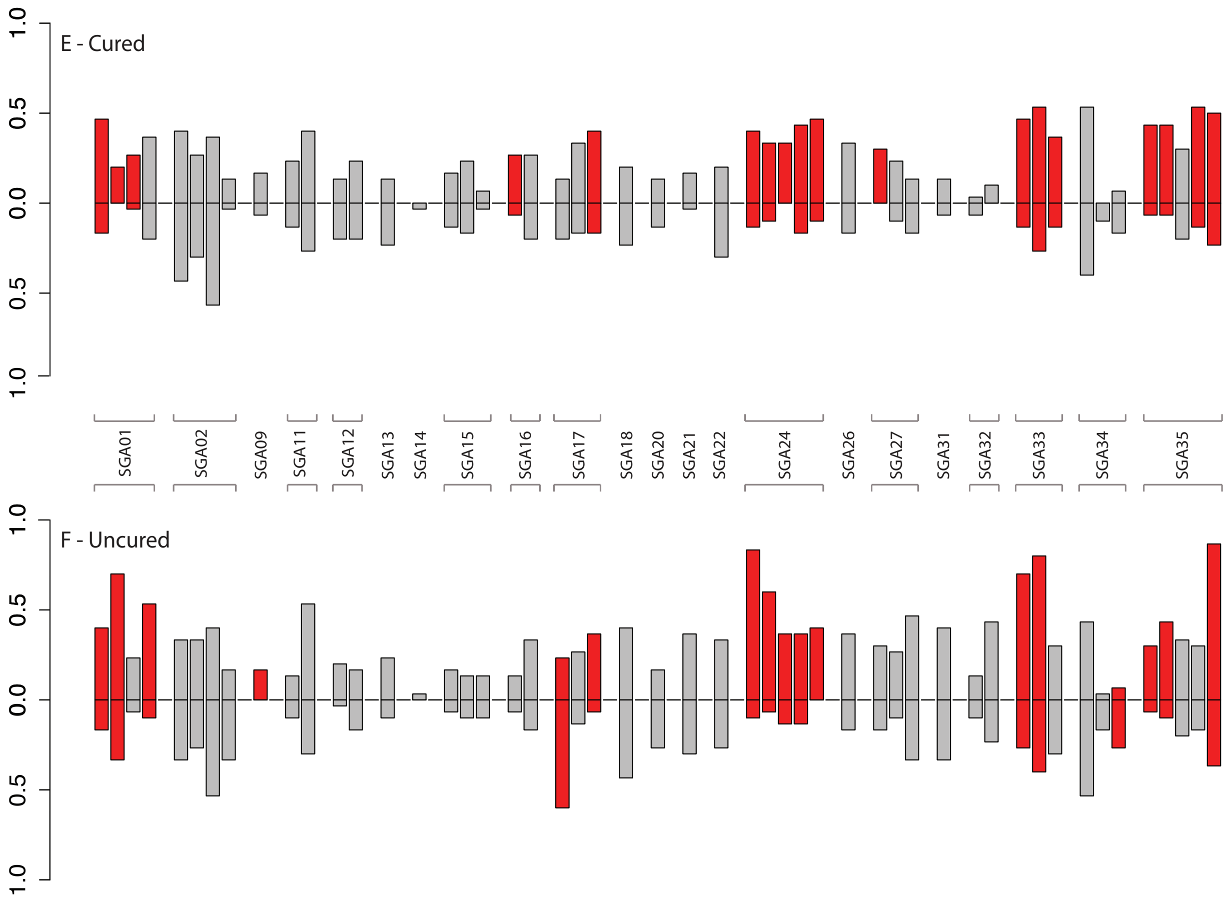

Figure S2. Hybrid dysgenic crosses and controls. Bar plots show the fraction of dysgenic offspring for both directions of each set of reciprocal crosses. Bars are coloured red when a significant difference between each reciprocal cross is found (Fisher's Exact Test, $p < 0.05$). **A.** Initial crosses between Georgia (2009) and Madagascar (2004) flies at 29° C, with the fraction of dysgenic F1 females shown when the Georgia strain is paternal (positive direction) or maternal (negative direction). **B.** A subset of the crosses in panel A were repeated at 25° C, a non-dysgenic temperature in *D. melanogaster*; repeated crosses are shown in the same position as in panel A. **C.** Fraction of dysgenic offspring from crosses within and between Georgia and Florida lines at 29° C. The first strain named in the cross is the paternal strain in the positive direction and the maternal strain in the negative direction. **D.** Fraction of dysgenic offspring from crosses within and between Madagascar lines at 29° C. The first strain named in the cross is the paternal strain in the positive direction and the maternal strain in the negative direction. **E.** Fraction of dysgenic offspring from crosses between Georgia and Madagascar lines at 29C after curing of wolbachia with tetracycline hydrachloride for 2 generations. **F.** Fraction of dysgenic offspring from crosses between Georgia and Madagascar lines at 29C before curing.
