# Supplementary material for: Hybrid Dysgenesis in Drosophila simulans Associated with a Rapid Invasion of the P-Element
Source: PLoS Genet. 2016 Mar 16;12(3):e1005920. doi: 10.1371/journal.pgen.1005920 (PMC4794157; doi:10.1371/journal.pgen.1005920)
Supplement: S3 Fig — Map shows the approximate location where strains were collected; pie charts show the proportion of each population which were dysgenesis-inducing (red), dysgenesis-resistant (orange) and dysgenesis-susceptible (yellow). The area of the pie chart is proportional to the number of strains sampled. For raw data see: http://dx.doi.org/10.5061/dryad.1rq8f. (PDF) [file pgen.1005920.s003.pdf]

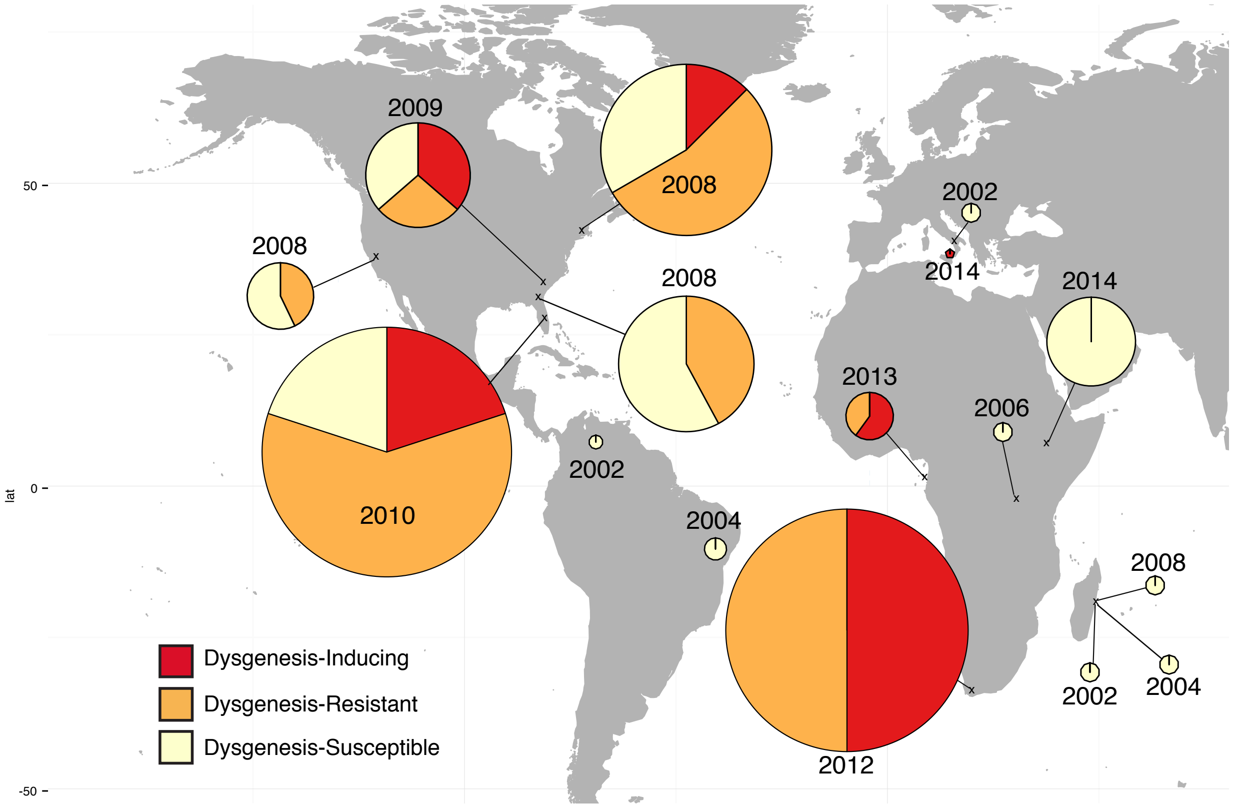

Figure S3. Map shows the approximate location where strains were collected; pie charts show the proportion of each population which were dysgenesis-inducing (red), dysgenesis-resistant (orange) and dysgenesis-susceptible (yellow). This summarizes the data given in Table 2. The area of the pie chart is proportional to the number of strains sampled.
