# Supplementary material for: Hybrid Dysgenesis in Drosophila simulans Associated with a Rapid Invasion of the P-Element
Source: PLoS Genet. 2016 Mar 16;12(3):e1005920. doi: 10.1371/journal.pgen.1005920 (PMC4794157; doi:10.1371/journal.pgen.1005920)
Supplement: S4 Fig — Plots A-C show the number of sterile F1 offspring produced from the dysgenic (red) and reciprocal (grey) crosses (from 35 total offspring), shown with 95% confidence interval. Crosses that show a significant difference in sterility between directions are marked with a star (Fisher’s Exact Test p < 0.05). Plots D-F show the number of offspring produced by the female offspring from both the dysgenic and reciprocal cross. Crosses that show a significant difference in the number of offspring for each direction are marked with a star (Wilcox Rank Sum Test p < 0.05). Female parents were dissected after crossing to confirm the presence of gonadal dysgenesis (mean of 50.6% of female parents were dysgenic, compared to a mean of 8.6% dysgenic female parents in the reciprocal cross, note this is a similar proportion to the proportion of sterile F1 females). A & D. Females were mated after 3–9 days of aging. B & E. Females were mated after 10–16 days of aging. C & F. Combined results for all crosses and ages. (PDF) [file pgen.1005920.s004.pdf]

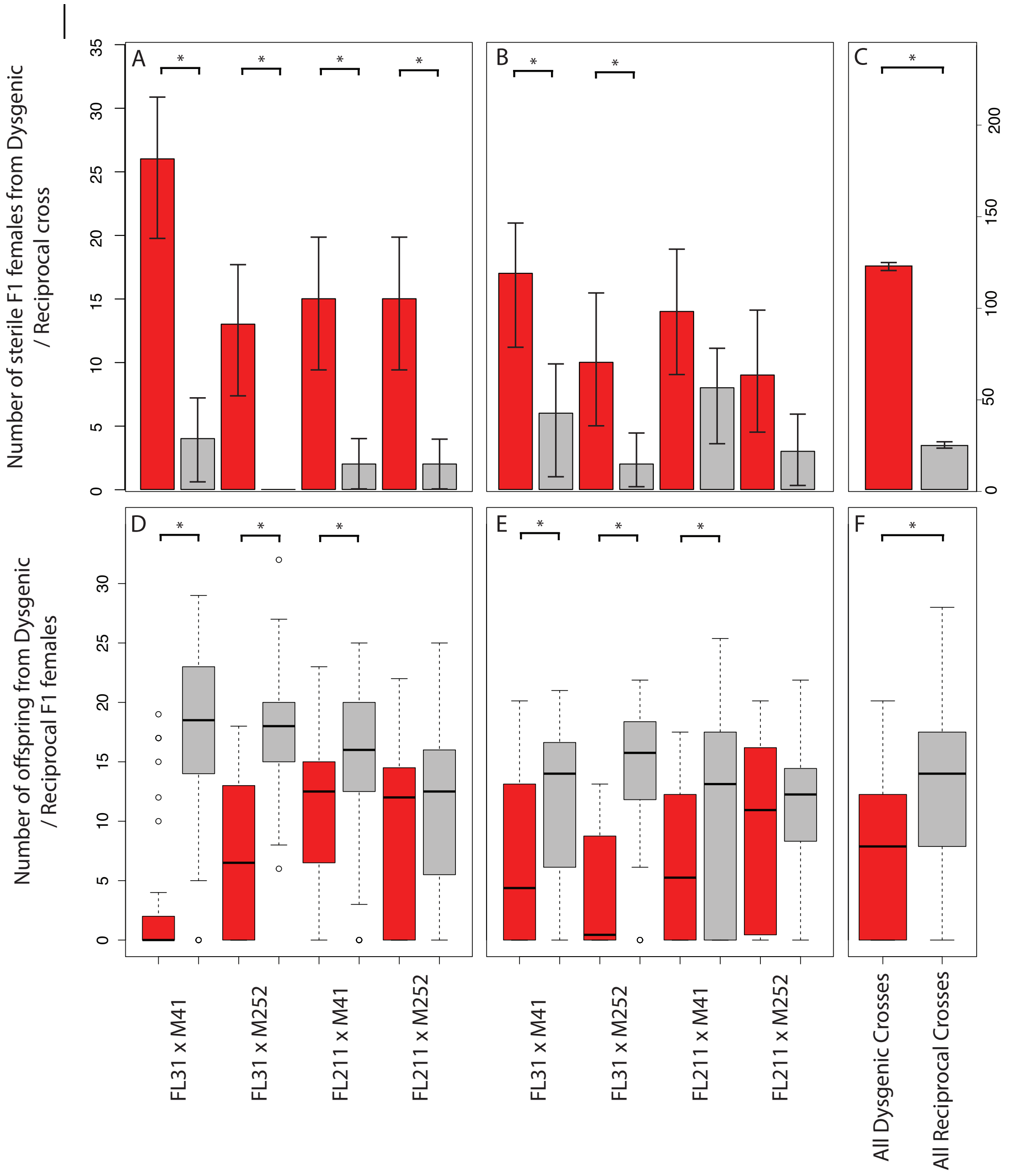

Figure S4. Plots **A-C** show the number of sterile F1 offspring produced from the dysgenic (red) and reciprocal (grey) crosses (from 35 total offspring), shown with 95% confidence interval. Crosses that show a significant difference in sterility between directions are marked with a star (Fisher's Exact Test $p < 0.05$). Plots **D-F** show the number of offspring produced by the female offspring from both the dysgenic and reciprocal cross. Crosses that show a significant difference in the number of offspring for each direction are marked with a star (Wilcox Rank Sum Test $p < 0.05$). Female parents were dissected after crossing to confirm the presence of gonadal dysgenesis (mean of 50.6% of female parents were dysgenic, compared to a mean of 8.6% dysgenic female parents in the reciprocal cross, note this is a similar proportion to the proportion of sterile F1 females). **A & D.** Females were mated after 3-9 days of aging. **B & E.** Females were mated after 10-16 days of aging. **C & F.** Combined results for all crosses and ages.
