# Supplementary material for: Hybrid Dysgenesis in Drosophila simulans Associated with a Rapid Invasion of the P-Element
Source: PLoS Genet. 2016 Mar 16;12(3):e1005920. doi: 10.1371/journal.pgen.1005920 (PMC4794157; doi:10.1371/journal.pgen.1005920)
Supplement: S5 Fig — Average coverage for TEs in the sequenced Florida (2010) x Madagascar (2004) isofemale lines vs. coverage for TEs in M252 (2004). Here, coverage is defined as the average per base coverage of TE sequence, divided by the average per base coverage of chromosome 2R in the line. The Florida coverage is taken as the average coverage of 12 available lines (FL3, FL6, FL98, FL101, FL116, FL136, FL168, FL174, FL189, FL198, FL208 & FL211). The Florida data (SRA:PRJEB7936, PRJNA308281) and Madagascar data (SRA:SRX504933) was mapped to the D. simulans reference genome alongside Flybase TE sequences to identify differences in coverage for these TEs. Only one TE was found in Florida but not in Madagascar, P-element. (PDF) [file pgen.1005920.s005.pdf]

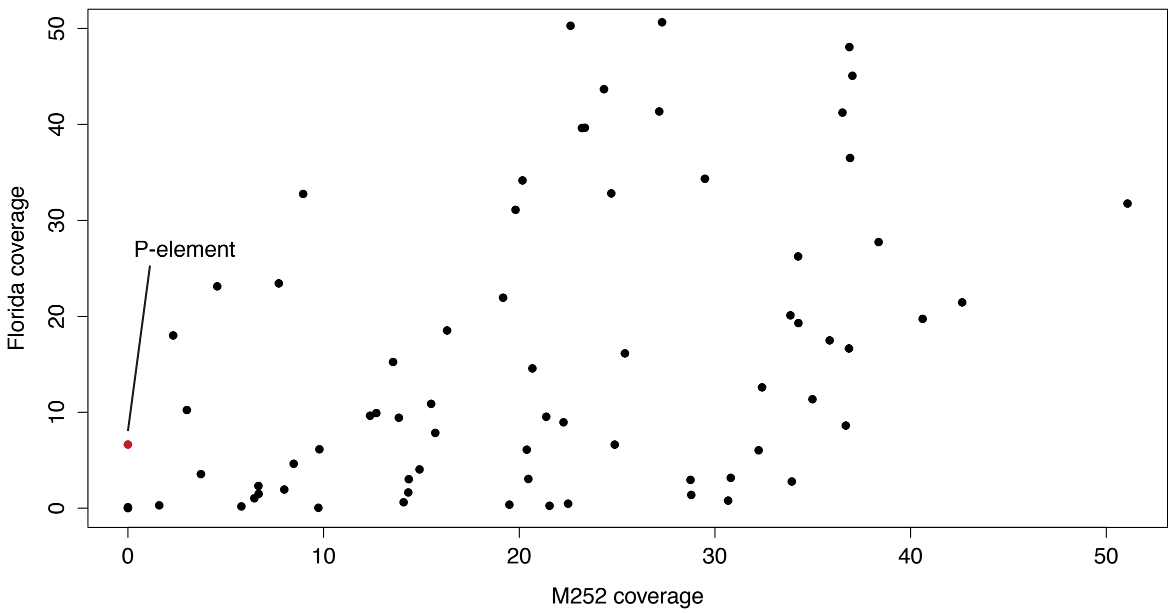

Figure S5. Average coverage for TEs in the sequenced Florida (2010) x Madagascar (2004) isofemale lines *vs.* coverage for TEs in M252 (2004). Here, coverage is defined as the average per base coverage of TE sequence, divided by the average per base coverage of chromosome 2R in the line. The Florida coverage is taken as the average coverage of 12 available lines (FL3, FL6, FL98, FL101, FL116, FL136, FL168, FL174, FL189, FL198, FL208 & FL211). The Florida data (SRA:PRJEB7936) and Madagascar data (SRA:SRX504933) was mapped to the *D. simulans* reference genome alongside Flybase TE sequences to identify differences in coverage for these TEs. Only one TE was found in Florida but not in Madagascar, P-element.
