# Supplementary material for: Hybrid Dysgenesis in Drosophila simulans Associated with a Rapid Invasion of the P-Element
Source: PLoS Genet. 2016 Mar 16;12(3):e1005920. doi: 10.1371/journal.pgen.1005920 (PMC4794157; doi:10.1371/journal.pgen.1005920)
Supplement: S6 Fig — Estimated copy number of TE (estimated via dividing the average coverage of the TE in a sample by the average coverage of chromosome 2R) vs. the fraction of dysgenic offspring observed in a cross (12 lines from Florida population [described in S5 Fig] crossed to M26 [in red] and M252 [in black]), with lines showing the fit of a binomial generalised linear model. A) The correlation between the copy number of P-element and the proportion of dysgenic offspring from the paternal Florida crosses (S2 Table; z value = 6.49, p = 8.49e-11). The equivalent association between dysgenesis and P-element copy number for D. melanogaster is shown with clear points [16]. B) The correlation between the copy number of P-element and the proportion of dysgenic offspring from the maternal Florida crosses (S2 Table; z value = -1.128, p = 0.259). C. The correlation between the copy number of Tom1 and the proportion of dysgenic offspring from the paternal Florida crosses (S2 Table; z value = -0.551, p = 0.581). D. The correlation between the copy number of Tom1 and the proportion of dysgenic offspring from the maternal Florida crosses (S2 Table; z value = 0.135, p = 0.893). (PDF) [file pgen.1005920.s006.pdf]

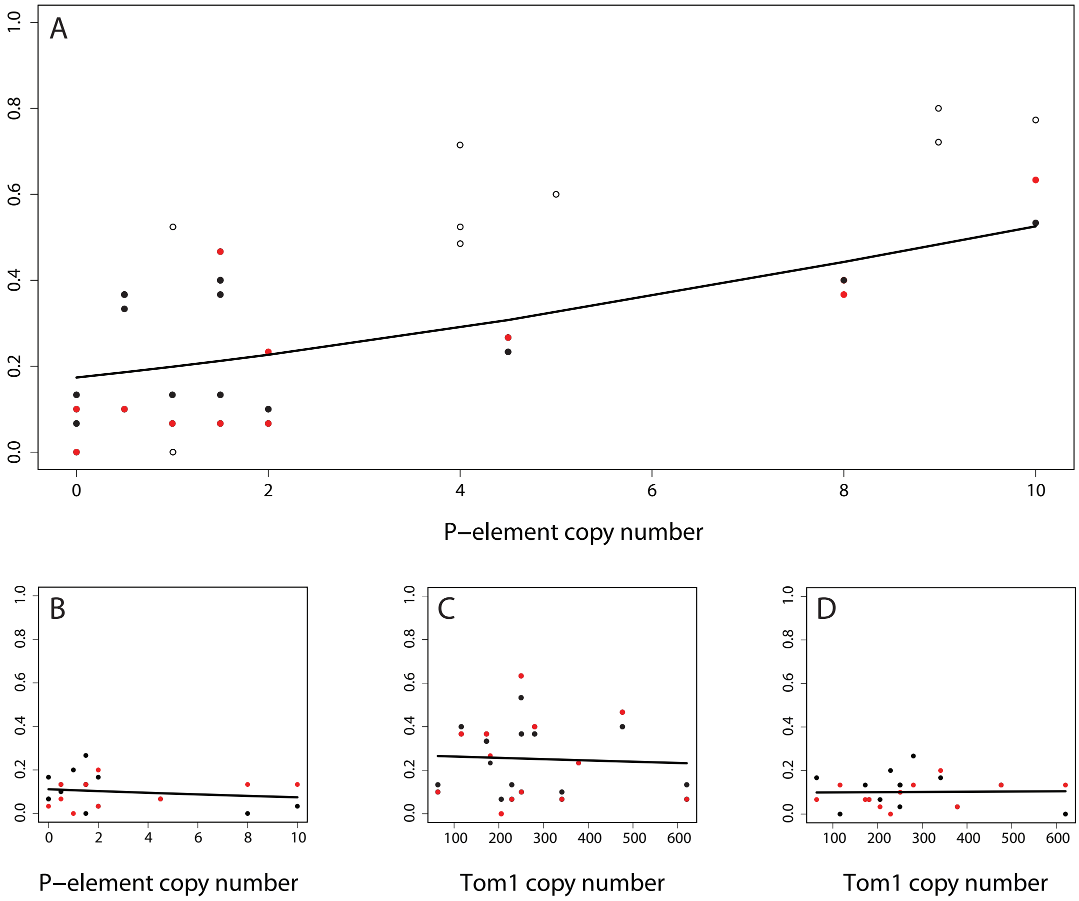

Figure S6. Estimated copy number of TE (estimated via dividing the average coverage of the TE in a sample by the average coverage of chromosome 2R) *vs.* the fraction of dysgenic offspring observed in a cross (12 lines from Florida population [described in Figure S5] crossed to M26 [in red] and M252 [in black]), with lines showing the fit of a binomial generalised linear model. **A.** The correlation between the copy number of P-element and the proportion of dysgenic offspring from the paternal Florida crosses (Supplementary Table 2; z value = 6.49, p =8.49e-11). The equivalent association between dysgenesis and P-element copy number for *D. melanogaster* is shown with clear points (Bingham *et al*, 1982). **B.** The correlation between the copy number of P-element and the proportion of dysgenic offspring from the maternal Florida crosses (Supplementary Table 2; z value = -1.128, p = 0.259). **C.** The correlation between the copy number of Tom1 and the proportion of dysgenic offspring from the paternal Florida crosses (Supplementary Table 2; z value = -0.551, p =0.581). **D.** The correlation between the copy number of Tom1 and the proportion of dysgenic offspring from the maternal Florida crosses (Supplementary Table 2; z value = 0.135, p =0.893).
