# Supplementary material for: Hybrid Dysgenesis in Drosophila simulans Associated with a Rapid Invasion of the P-Element
Source: PLoS Genet. 2016 Mar 16;12(3):e1005920. doi: 10.1371/journal.pgen.1005920 (PMC4794157; doi:10.1371/journal.pgen.1005920)
Supplement: S7 Fig — A) RT-PCR product from DI and DS lines. Group 1 consists of female flies from three DI lines roughly dissected into soma (S) and ovaries (G). The top band, at 2.6kb, corresponds to the expected size of the full-length P-element transcript after splicing. Group 2 consists of female flies from two DS lines with a positive control (D. melanogaster Harwich strain). B) PCR product from DI, DR & DS lines following reverse transcription with either forward (1) or reverse (2) primers. (PDF) [file pgen.1005920.s007.pdf]

A.

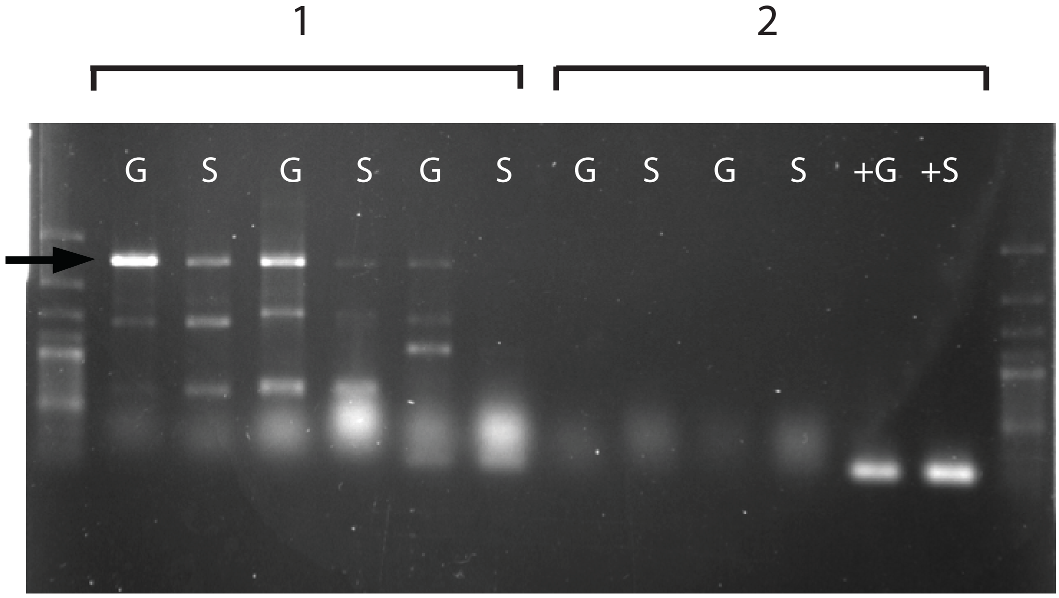

B.

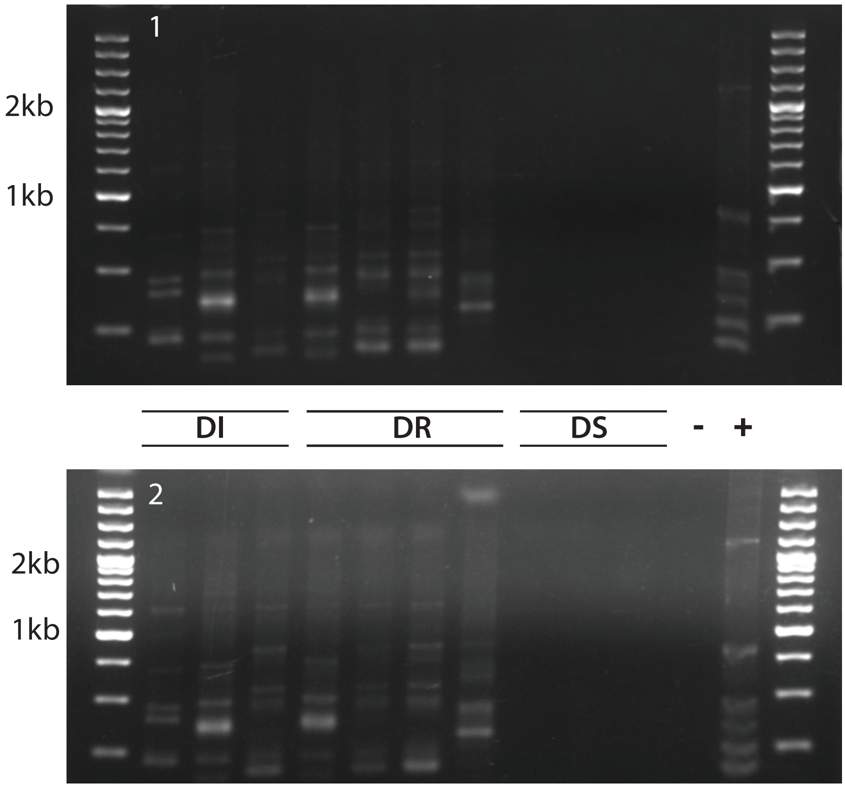

Figure S7. **A.** RT-PCR product from DI and DS lines. Group 1 consists of female flies from three DI lines roughly dissected into soma (S) and ovaries (G). The top band, at 2.6kb, corresponds to the expected size of the full-length P-element transcript after splicing. Group 2 consists of female flies from two DS lines with a positive control (*D. melanogaster* Harwich strain). **B.** PCR product from DI, DR & DS lines following reverse transcription with either forward (1) or reverse (2) primers.
