# Supplementary material for: Hybrid Dysgenesis in Drosophila simulans Associated with a Rapid Invasion of the P-Element
Source: PLoS Genet. 2016 Mar 16;12(3):e1005920. doi: 10.1371/journal.pgen.1005920 (PMC4794157; doi:10.1371/journal.pgen.1005920)
Supplement: S1 Table — Table includes time and date of collection, and collector. The fraction of strains with P-element are also shown for each collection. (PDF) [file pgen.1005920.s008.pdf]

Table S1. Fly strains used in this study, including time and date of collection, and collector. The fraction of strains with P-element are shown for each collection.

						Proportion of strains with P-element exon				Number of strains with P-element			
**Population**	**n**	**species**	**Location**	**Year**	**Collector**	**exon 0**	**exon 1**	**exon 2**	**exon 3**	**full**	**partial**	**none**	**total**
SGA	22	*D. simulans*	Athens, Georgia, USA	2009	P.Haddrill	0.68	0.5	0.45	1	7	15	0	22
ANDA	7	*D. simulans*	Andasibe, Madagascar	2008	J.R. David	0.56	0.84	0.14	0.28	0	6	1	7
M	5	*D. simulans*	unknown, Madagascar	2004	B. Ballard	0	0	0	0	0	0	5	5
Mad	1	*D. simulans*	unknown, Madagascar	2009	E. Helmich	1	0	0	0	0	1	0	1
TANA	7	*D. simulans*	Antananarivo, Madagascar	2008	J.R. David	0.42	0.42	0.14	0.71	0	5	2	7
FL	36	*D. simulans*	Tampa,Florida, USA	2010	R. Tobler	0.81	0.56	0.56	0.81	17	12	7	36
SA	34	*D. simulans*	Kanonkop, South Africa	2013	H. van Schalkwyk	1	1	1	1	34	0	0	34
HAR	14	*D. simulans*	Harvard,Massachussetts, USA	2008	A. Paaby	0.28	0	0	0.14	0	4	10	14
DAV	19	*D. simulans*	Davis,California, USA	2009	M. Turelli	0.05	0.05	0	0	0	1	18	19
MED	17	*D. simulans*	Media,Pennsylvania, USA	2008	A. Paaby	0.12	0.059	0	0	0	2	15	17
CHU	13	*D. simulans*	Churchville,Maryland, USA	2008	A. Paaby	0.69	0.31	0.23	0.69	5	4	4	13
HIN	19	*D. simulans*	South Carolina, USA	2008	A. Paaby	0.053	0.053	0.053	0.053	0	1	18	19
IRF	13	*D. simulans*	Jasper, Florida, USA	2008	A. Paaby	0.077	0.077	0.077	0	0	2	11	13
LPS	10	*D. simulans*	Morven,Georgia, USA	2008	A. Paaby	0	0	0	0	0	0	10	10
PD	12	*D. simulans*	Padova, Italy	2002	E. Tauber	0	0	0	0	0	0	12	12
BGT	2	*D. simulans*	Guaymaral, close to Bogota, Columbia	2002	P. Orozco-terWengel	0	0	0	0	0	0	2	2
TU	1	*D. simulans*	Djerba, Tunisia	2001	M. Puchinger	0	0	0	0	0	0	1	1
ZOM	2	*D. simulans*	Zomba, Malawi	2001	C. Niessinger	0	0	0	0	0	0	2	2
KIB	2	*D. simulans*	Kibale, Uganda	2001	M. Imhof	0	0	0	0	0	0	2	2
ABU	9	*D. simulans*	Ubatuba, Brazil	2004	J. F. Garcia	0	0	0	0	0	0	9	9
Mandra	6	*D. simulans*	Mandraka Park, Madagscar	2008	J.R. David	0.67	0.67	0	0.5	0	4	2	6
YA	3	*D. simulans*	Nairobi, Kenya	2013	D.Matute	0.33	0.33	0	0.33	0	3	0	3
ST	7	*D. simulans*	Sao Tome, Sao Tome and Principe	2013	D.Matute	1	1	1	1	7	0	0	7
BS	5	*D. simulans*	Antananarivo, Madagscar	2013	D.Matute	1	1	1	1	5	0	0	5
BIO	15	*D. simulans*	Bioko Norte, Equatorial Guinea	2013	D.Matute	1	1	1	1	15	0	0	15
SREN	3	*D. simulans*	Sorrento, Italy	2014	M.Pegoraro	1	0.33	0.33	0.66	1	2	0	3
MD	16	*D. simulans*	Antananarivo, Madagscar	2002	B. Ballard	0	0	0	0	0	0	16	16
NS	12	*D. simulans*	Nairobi, Kenya	2006	B. Ballard	0	0	0	0	0	0	12	12
PORT	60	*D. simulans*	North Portugal, Portugal	2006	E.Sucena	0.13	0.18	0.16	0.18	3	10	47	60
POR	36	*D. simulans*	North Portugal, Portugal	2013	E.Sucena	1	1	1	1	36	0	0	36
IT	38	*D. simulans*	Bologna, Italy	2014	M.F.Shou	1	1	1	1	38	0	0	38
CRO	50	*D. simulans*	Zabgreb, Croatia	2014	A.M. Jaksic	1	1	1	1	50	0	0	50
ED	1	*D. simulans*	Dodola, Ethiopia	2008	J.Pool	1	0	0	1	0	1	0	1
KN	1	*D. simulans*	Nyahururu, Kenya	2009	J.Pool	1	0	0	1	0	1	0	1
UK	1	*D. simulans*	Kisoro, Uganda	2012	R.Corbett	1	0	0	1	0	1	0	1
UB	1	*D. simulans*	Bundibugyo, Uganda	2012	R.Corbett	1	0	0	1	0	1	0	1
TZ	1	*D. simulans*	Uyole, Tanzania	2009	L.Nsemwa	0	0	0	0	0	0	1	1
KR	1	*D. simulans*	Marigat, Kenya	2009	J.Pool	0	0	0	0	0	0	1	1
EZ	1	*D. simulans*	Ziway, Ethiopia	2008	J.Pool	0	0	0	0	0	0	1	1
SP	1	*D. simulans*	Phalaborwa, South Africa	2010	J.Pool	1	1	1	1	1	0	0	1
KT	1	*D. simulans*	Thika, Kenya	2009	J.Pool	1	0	0	1	0	1	0	1
BA	4	*D. simulans*	Puglia, Italy	2014	O.Rota-Stabelli	1	1	1	1	4	0	0	4
RIV	2	*D. simulans*	Riverside,California, USA	1988	M.Turelli	0	0	0	0	0	0	2	2
YOL	2	*D. simulans*	Yolo,California, USA	2010	M.Turelli	0	0.5	0	0	0	1	1	2
IRV	1	*D. simulans*	Irvine,California, USA	2014	M.Turelli	1	1	1	1	2	0	0	2
Mex1	1	*D. simulans*	Oaxaca, Mexico	2002	S.Castrezana	0	0	0	0	0	0	1	1
Mex2	1	*D. simulans*	Sonora, Mexico	2009	T.Markow	0	0	0	0	0	0	1	1
Mex3	1	*D. simulans*	Guanajuato, Mexico	2014	T.Markow	1	1	1	1	1	0	0	1
Mex4	1	*D. simulans*	Cusco, Peru	2009	D.Lindsley	0	1	0	0	0	1	0	1
CAM1	10	*D. simulans*	Nyasoso, Cameroon	2004	M. Vieulle	0	0	0	0	0	0	10	10
CAM2	25	*D. simulans*	Nyasoso, Cameroon	2007	M. Vieulle	0	0	0	0	0	0	25	25
CAM3	18	*D. simulans*	Nyasoso, Cameroon	2010	M. Vieulle	0	0	0	0	0	0	18	18
TANZ	41	*D. simulans*	Lignees, Tanzania	1996	M. Vieulle	0	0	0	0	0	0	41	41
ZIMB	6	*D. simulans*	Nyanga, Zimbabwe	1997	M. Vieulle	0	0	0	0	0	0	6	6
AA	11	*D. simulans*	Addis Ababa, Ethiopia	2014	W.Miller	0	0	0	0	0	0	12	12
LD	10	*D. sechellia*	La Digue, The Seychelles	2014	D.Matute	0	0	0	0	0	0	10	10
DNF	11	*D. sechellia*	Denis, The Seychelles	2014	D.Matute	0	0	0	0	0	0	11	11
ANRO	9	*D. sechellia*	Mahé, The Seychelles	2014	D.Matute	0	0	0	0	0	0	9	9
