# Supplementary material for: Hybrid Dysgenesis in Drosophila simulans Associated with a Rapid Invasion of the P-Element
Source: PLoS Genet. 2016 Mar 16;12(3):e1005920. doi: 10.1371/journal.pgen.1005920 (PMC4794157; doi:10.1371/journal.pgen.1005920)
Supplement: S2 Table — Number of reads mapping shows the number of reads which mapped to the TE in the expression data (SRA:PRJEB7936). The Z-value and P-value are from a binomial generalised linear model used to analyze the association between the TE copy number (found in the Florida sequence data, SRA:PRJEB7936, PRJNA308281) and the number of hybrid dysgenesis seen in a reciprocal crosses to an M line (see also S6 Fig); TEs causing the dysgenesis would be expected to show a significant relationship between dysgenesis in the cross where the Florida line is the male parent. Also shown is the number of DI strains and DS strains from which a TE could be amplified. Note that individual exons for the P-element could be amplified from all 39 DI strains; the results shown here are for amplification of the full- length P-element. (PDF) [file pgen.1005920.s009.pdf]

Table S2. Association between cytotypes of *D. simulans* strains and infection status for individual TE families. Number of reads mapping shows the number of reads which mapped to the TE in the expression data (SRA:PRJEB7936). The Z-value and P-value are from a binomial generalised linear model used to analyze the assocation betweem the TE copy number (found in the Florida sequence data, SRA:PRJEB7936) and the number of hybrid dysgenesis seen in an reciporical crosses to an M line (see also Figure S6); TEs causing the dysgenesis would be expected to show a significant relationship between dysgenesis in the cross where the Florida line is the male parent. Also shown is the number of DI strains and DS strains from which a TE could be amplified. Note that individual exons for the P-element could be amplified from all 39 DI strains; the results shown here are for amplification of the full-length P-element.

**FlyBase Accession**	**TE name**	**NCBI Accession**	**No. of Reads Mapping to TE**	**Florida Male x M Female - Linear Regression**		**Florida Female x M Male - Linear Regression**		**DI strains**		**DS strain**		**FET p-value**
				Z value	p-value	Z value	p-value	present	absent	present	absent	
FBgn0000004	17.6	X01472	2	-1.185	0.236	0.004	0.997	39	0	78	0	1
FBgn0000005	297	X03431	130	-0.254	0.800	-0.307	0.759	39	0	78	0	1
FBgn0000007	1731	X07656	1430	-0.411	0.681	-0.975	0.330	39	0	78	0	1
FBgn0000155	roo	AY180917	2046	1.111	0.267	0.357	0.721	39	0	78	0	1
FBgn0000199	blood	AY180916	690	-1.049	0.294	-0.143	0.886	39	0	78	0	1
FBgn0000349	copia	X02599	162	-0.363	-0.363	0.716	-0.182	39	0	78	0	1
FBgn0000481	Doc	X17551	668	0.891	0.373	-0.290	0.772	39	0	78	0	1
FBgn0000638	FB	V00246	62	2.183	0.029	0.552	0.581	39	0	78	0	1
FBgn0000652	F-element	AC005198	66	1.049	0.294	0.824	0.410	39	0	78	0	1
FBgn0001100	G-element	X06950	2	-0.859	-0.859	0.390	-0.451	39	0	78	0	1
FBgn0001167	gypsy	M12927	82	0.044	0.965	-0.612	0.541	39	0	78	0	1
FBgn0001181	HB	X01748	36	0.957	0.339	-0.164	0.869	39	0	78	0	1
FBgn0001207	HMS-Beagle	AF365402	222	-0.085	0.932	-0.873	0.383	39	0	78	0	1
FBgn0001210	hobo	M69216	1070	1.842	0.066	0.093	0.926	39	0	78	0	1
FBgn0001249	I-element	M14954	234	0.975	0.329	-0.333	0.739	39	0	78	0	1
FBgn0001283	jockey	M22874	56	0.077	0.938	-0.520	0.603	39	0	78	0	1
FBgn0002651	Dmau\mariner	M14653	356	-2.321	0.728	-1.042	0.930504	39	0	78	0	1
FBgn0002697	mdg1	X59545	108	-0.037	0.971	-0.101	0.920	39	0	78	0	1
FBgn0002698	mdg3	X95908	116	-0.583	0.560	-1.015	0.310	39	0	78	0	1
FBgn0002745	micropia	X14037	40	0.320	0.749	-0.308	0.758	39	0	78	0	1
FBgn0003007	opus	AY180918	1226	-0.338	0.735	-0.003	0.998	39	0	78	0	1
**FBgn0003055**	**P-element**	**X06779**	**1220**	**6.492**	**8.49E-11**	**-1.128**	**0.259**	**33**	**6**	**0**	**78**	**2.37E-23**
FBgn0003519	Stalker	AF420242	18	-0.288	0.774	0.059	0.953	39	0	78	0	1
FBgn0003908	R1A1-element	X51968	208	0.965	0.334	0.094	0.925	39	0	78	0	1
FBgn0003909	R2-element	X51967	120	1.248	0.212	0.008	0.994	39	0	78	0	1
FBgn0004082	Tirant	X93507	0	-0.733	0.464	-0.144	0.885	39	0	78	0	1
FBgn0004141	HeT-A	U06920	4	-1.625	0.104	-1.147	0.251	39	0	78	0	1
FBgn0004904	TART-A	AY561850	8684	-1.604	0.109	1.417	0.156	39	0	78	0	1
FBgn0004904	TART-B	U14101	2996	-0.702	0.483	1.008	0.313	39	0	78	0	1
FBgn0005384	3S18	U23420	124	-0.064	0.949	0.148	0.882	39	0	78	0	1
FBgn0005673	1360	AC005453	1873	0.494	0.621	-0.360	0.719	39	0	78	0	1
FBgn0005773	Bari1	X67681	786	1.597	0.110	0.151	0.880	39	0	78	0	1
FBgn0010103	aurora-element	AB022762	68	-0.379	0.704	-0.694	0.488	39	0	78	0	1
FBgn0010302	Burdock	U89994	808	0.221	0.825	-0.790	0.429	39	0	78	0	1
FBgn0013017	Dtei\I-element	M28878	2	-1.219	0.874	-0.826	0.953519	39	0	78	0	1
FBgn0013099	Dvir\Tv1	AF056940	1	-1.332	0.710	-0.892	0.896221	39	0	78	0	1
FBgn0014947	flea	Z27119	390	-0.595	0.552	0.297	0.767	39	0	78	0	1
FBgn0014967	hopper	X80025	114	0.750	0.454	0.174	0.862	39	0	78	0	1
FBgn0015168	Dsim\ninja	D83207	1246	-1.91	0.448	-1.519	0.803856	39	0	78	0	1
FBgn0015945	GATE	AJ010298	788	-0.028	0.977	-0.344	0.731	39	0	78	0	1
FBgn0023131	ZAM	AJ000387	58	-0.435	0.664	-0.008	0.994	39	0	78	0	1
FBgn0026065	Idefix	AJ009736	2	-0.522	0.601	-0.523	0.601	39	0	78	0	1
FBgn0026416	INE-1	U66884	8	0.903	0.367	-0.375	0.708	39	0	78	0	1
FBgn0026443	Dyak\TART	AF468026	152	-0.865	0.519	-0.804	0.638228	39	0	78	0	1
FBgn0040267	Transpac	AF222049	204	-0.768	0.442	-0.684	0.494	39	0	78	0	1
FBgn0041728	Rt1a	AJ278684	16	-0.769	0.442	-0.037	0.971	39	0	78	0	1
FBgn0042231	X-element	AF237761	536	1.072	0.284	-0.073	0.942	39	0	78	0	1
FBgn0042682	Rt1b	AF281636	642	0.070	0.944	-0.232	0.817	39	0	78	0	1
FBgn0044997	Dfun\Isfun-1	AJ309320	1	-1.852	0.930	-1.233	0.927757	39	0	78	0	1
FBgn0046110	Juan	AY180919	1111	0.501	0.616	-0.573	0.567	39	0	78	0	1
FBgn0063432	gypsy5	AE003485	69	-0.446	0.656	-1.002	0.316	39	0	78	0	1
FBgn0063434	gypsy3	AC007477	301	-0.005	0.996	-0.583	0.560	39	0	78	0	1
FBgn0063435	gypsy2	AL035631	100	-0.372	0.710	-0.301	0.763	39	0	78	0	1
**FBgn0063450**	**Tom1**	**Z24451**	**118**	**-0.551**	**0.581**	**0.135**	**0.893**	**39**	**0**	**71**	**7**	**0.0935738**
FBgn0063755	Osvaldo	AY089271	172	-0.075	0.940	-0.467	0.641	39	0	78	0	1
FBgn0063782	accord2	AF541947	464	-0.069	0.945	-0.206	0.837	39	0	78	0	1
FBgn0063897	Stalker4	AF541949	16	-0.451	0.652	-0.109	0.913	39	0	78	0	1
FBgn0063917	McClintock	AF541948	76	-0.009	0.993	-0.394	0.693	39	0	78	0	1
FBgn0063919	Max-element	AJ487856	8042	0.659	0.510	-0.313	0.754	39	0	78	0	1
FBgn0066148	Dvir\TART	AY219709	52	-1.362	0.013	0.3357	0.00176	39	0	78	0	1
FBgn0067380	invader6	NT_033778	788	-0.120	0.904	0.002	0.999	39	0	78	0	1

FlyBase Accession	TE name	NCBI Accession	No. of Reads Mapping to TE	Florida Male x M Female - Linear Regression		Florida Female x M Male - Linear Regression		DI strains		DS strain		FET p-value
				Z value	p-value	Z value	p-value	present	absent	present	absent	
FBgn0000004	17.6	X01472	2	-1.185	0.236	0.004	0.997	39	0	78	0	1
FBgn0000005	297	X03431	130	-0.254	0.800	-0.307	0.759	39	0	78	0	1
FBgn0000007	1731	X07656	1430	-0.411	0.681	-0.975	0.330	39	0	78	0	1
FBgn0000155	roo	AY180917	2046	1.111	0.267	0.357	0.721	39	0	78	0	1
FBgn0000199	blood	AY180916	690	-1.049	0.294	-0.143	0.886	39	0	78	0	1
FBgn0000349	copia	X02599	162	-0.363	-0.363	0.716	-0.182	39	0	78	0	1
FBgn0000481	Doc	X17551	668	0.891	0.373	-0.290	0.772	39	0	78	0	1
FBgn0000638	FB	V00246	62	2.183	0.029	0.552	0.581	39	0	78	0	1
FBgn0000652	F-element	AC005198	66	1.049	0.294	0.824	0.410	39	0	78	0	1
FBgn0001100	G-element	X06950	2	-0.859	-0.859	0.390	-0.451	39	0	78	0	1
FBgn0001167	gypsy	M12927	82	0.044	0.965	-0.612	0.541	39	0	78	0	1
FBgn0001181	HB	X01748	36	0.957	0.339	-0.164	0.869	39	0	78	0	1
FBgn0001207	HMS-Beagle	AF365402	222	-0.085	0.932	-0.873	0.383	39	0	78	0	1
FBgn0001210	hobo	M69216	1070	1.842	0.066	0.093	0.926	39	0	78	0	1
FBgn0001249	I-element	M14954	234	0.975	0.329	-0.333	0.739	39	0	78	0	1
FBgn0001283	jockey	M22874	56	0.077	0.938	-0.520	0.603	39	0	78	0	1
FBgn0002651	Dmau\mariner	M14653	356	-2.321	0.728	-1.042	0.930504	39	0	78	0	1
FBgn0002697	mdg1	X59545	108	-0.037	0.971	-0.101	0.920	39	0	78	0	1
FBgn0002698	mdg3	X95908	116	-0.583	0.560	-1.015	0.310	39	0	78	0	1
FBgn0067381	hopper2	AF541950	2	0.113	0.910	0.192	0.848	39	0	78	0	1
FBgn0067382	gypsy9	AE002591	6	-0.700	0.484	0.373	0.709	39	0	78	0	1
FBgn0067383	gypsy8	AE003788	4	-0.571	0.568	-0.550	0.582	39	0	78	0	1
FBgn0067384	gypsy7	AE003788	4	-1.110	0.267	-0.339	0.735	39	0	78	0	1
FBgn0067385	gypsy12	AE003789	216	-0.619	0.536	-0.374	0.709	39	0	78	0	1
FBgnnnnnnnn	Dpse\mini-me	AC131959	1510	-1.302	0.683	-0.787	0.814311	39	0	78	0	1
