# Supplementary material for: Hybrid Dysgenesis in Drosophila simulans Associated with a Rapid Invasion of the P-Element
Source: PLoS Genet. 2016 Mar 16;12(3):e1005920. doi: 10.1371/journal.pgen.1005920 (PMC4794157; doi:10.1371/journal.pgen.1005920)
Supplement: S3 Table — The model used fit the year and region from which strains were collect (see S1 Table), plus an interaction term, to the presence or absence of P-element in that strain, and used a binomial error model. (i.e., the call in R glm(formula = cbind(present,absent) ~ year * region, family = "binomial") (PDF) [file pgen.1005920.s010.pdf]

Table S3. Generalized linear model fit to the data on PCR presence/absence data. The model used fit the year and region from which strains were collect (see Supplmentary Table 1), plus an interaction term, to the presence or absence of P-element in that strain, and used a binomial error model. (I.e, the call in R glm(formula = cbind(present,absent) ~ year * region, family = "binomial")

	odds-ratio estimate	std error	z	p-value	
(Intercept)	-9.11E+02	1.75E+02	-5.216	1.83E-07	***
year	4.53E-01	8.68E-02	5.218	1.81E-07	***
region(Americas)	-1.27E+03	4.76E+02	-2.664	0.00772	**
region(Europe)	-2.33E+02	2.24E+02	-1.041	0.29799	
interaction year x region(Americas)	6.31E-01	2.37E-01	2.663	0.00773	**
interaction year x region(Europe)	1.16E-01	1.11E-01	1.045	0.29625	

***p<0.001, **p<0.01
