# Supplementary material for: Hybrid Dysgenesis in Drosophila simulans Associated with a Rapid Invasion of the P-Element
Source: PLoS Genet. 2016 Mar 16;12(3):e1005920. doi: 10.1371/journal.pgen.1005920 (PMC4794157; doi:10.1371/journal.pgen.1005920)
Supplement: S4 Table — (PDF) [file pgen.1005920.s011.pdf]

Table S4. Primers used for identifying the presence or absence of P-element

Start position and direction	Sequence
Exon0 – 94 forward	GGTTGTGTGCGGACGAATTTT
Exon0 – 378 reverse	CTGGTTCAGGCTCTATCACTTT
Exon1 – 615 forward	TCTACGCAAAATCTTCACGGAC
Exon1 – 1144 reverse	CTGATATACCGAGCTCTGTCCA
Exon2 – 1241 forward	TCCTGCAGATGACCATTTAAAGA
Exon2 – 1900 reverse	TTAAACTGCAGTGGAGTGGGAT
Exon3 – 2181 forward	GGACAACTCTGAAAGCTGGC
Exon3 – 2545 reverse	CGTTTCGCGCTGCTAATATTAA
TIR – 3-31 forward & reverse	TGATGAAATAACATAAGGTGGTCCCGTCG
